# Predictors of thrombolysis in the telestroke and non telestroke settings for hypertensive acute ischemic stroke patients

**DOI:** 10.1186/s12883-018-1204-3

**Published:** 2018-12-21

**Authors:** Leanne Brecthel, Jordan Gainey, Alexandria Penwell, Thomas I. Nathaniel

**Affiliations:** University of South Carolina, School of Medicine-Greenville, Greenville, SC 29605 USA

**Keywords:** Acute ischemic stroke, Hypertension, rtPA, Inclusion criteria, Exclusion criteria

## Abstract

**Background:**

In acute ischemic stroke patients, telestroke technology provides sustainable approaches to improve the use of thrombolysis therapy. How this is achieved as it relates to inclusion or exclusion of clinical risk factors for thrombolysis is not fully understood. We investigated this in a population of hypertensive stroke patients.

**Methods:**

Structured data from a regional stroke registry that contained telestroke and non telestroke patients with a primary diagnosis of acute ischemic stroke with history of hypertension were collected between January 2014 and June 2016. Clinical risk factors associated with inclusion or exclusion for recombinant tissue plasminogen activator (rtPA) in the telestroke and non telestroke were identified using multiple regression analysis. Associations between variables and rtPA in the regression models were determined using variance inflation factors while the fitness of each model was determined using the ROC curve to predict the power of each logistic regression model.

**Results:**

The non telestroke admitted more patients (62% vs 38%), when compared with the telestroke. Although the telestroke admitted fewer patients, it excluded 11% and administered thrombolysis therapy to 89% of stroke patients with hypertension. In the non telestroke group, adjusted odd ratios showed significant associations of NIH stroke scale score (OR = 1.059, 95% CI, 1.025–1.093, *P* < 0.001) and coronary artery disease (OR = 2.003, 95% CI, 1.16–3.457, *P* = 0.013) with inclusion, while increasing age (OR = 0.979, 95% CI, 0.961–0.996, *P* = 0.017), higher INR (OR = 0.146, 95% CI, 0.032–0.665, *P* = 0.013), history of previous stroke (OR = 0.39, 95% CI, 0.223–0.68, *P* = 0.001), and renal insufficiency (OR = 0.153, 95% CI, 0.046–0.508, *P* = 0.002) were associated with rtPA exclusion. In the telestroke, only direct admission to the telestroke was associated with rtPA administration, (OR = 4.083, 95% CI, 1.322–12.611, *P* = 0.014).

**Conclusion:**

The direct admission of hypertensive stroke patients to the telestroke network was the only factor associated with inclusion for thrombolysis therapy even after adjustment for baseline variables. The telestroke technology provides less restrictive criteria for clinical risk factors associated with the inclusion of hypertensive stroke patients for thrombolysis.

## Background

One major attempt to address the rural-to-urban disparity and expand the availability of best stroke practices is the development of telestroke networks for acute stroke evaluation, management and clinical care [1–3]. Telestroke technology works to leverage the expertise of the stroke centers to extend the benefits of systemic thrombolysis to rural areas, and to eliminate inequities between rural and urban care of stroke patients. The number of patients receiving rtPA increases by almost a factor of ten over previous numbers when telestroke technology is applied [4, 5]. This is because telestroke extends the expertise of stroke centers to provide enhanced stroke care, particularly the administration of rtPA to smaller rural and community hospitals [6, 7]. Like all medical novelties, it is faster, employs the most recent technology and promotes a better use of limited resources.

Moreover, telestroke technology represents a major growth in many hospitals’ efforts to reduce door-to-needle time [3, 8] outside the traditional role of the telestroke in providing the hub-and-spoke system model for stroke care. Such a centralized role of the telestroke in reducing door-to-needle time may affect treatment times and use of thrombolysis [9–18]. Whether such a centralized role of the telestroke contributes to the reduction in pretreatment clinical risk factors associated with thrombolysis therapy is not very clear. Irrespective of whether the stroke patient is treated in the telestroke or non telestroke, hypertension is the most influential adjustable variable and the second most powerful clinical risk factor after age for stroke [15, 19–21]. Hypertensive patients are 60–70% more likely to suffer a stroke when compared to those without hypertension [22–25]. The strong relationship between stroke and hypertension has been investigated extensively [18, 23, 26–29]. Findings from these studies result in suggestions for the optimization of various hypertensive medications in hypertensive stroke patients prior to treatment with thrombolysis therapy. High-quality telestroke care remains efficient for all stroke patients [30], and the role of the telestroke is expected to increase with benefits of thrombolysis [31] for stroke patients including those with incidence of hypertension. Past studies have shown that decreases in mean arterial pressure (MAP) have a negative association with cerebral blood flow and patients with chronic hypertension have shifts in perfusion autoregulation parameters as well as changes in collateral blood supplies [32, 33]. This, in conjunction with other considerations such as carotid artery stenosis [34–36], put hypertensive stroke patients at a unique risk special consideration should be considered when determining rtPA eligibility in these patients.

In general, the association of the telestroke technology and thrombolysis therapy with exclusion or inclusion clinical risk factors in hypertensive stroke is not fully understood. Since stroke with hypertension is dynamic and complicated, one possibility is that the telestroke technology may be less or more stringent in the inclusion or exclusion of clinical risk factors for thrombolysis therapy when compared with the non telestroke setting for hypertensive stroke patients. We hypothesize that the practice-based model of telestroke can manage pretreatment clinical risk factors in hypertensive stroke patients for thrombolysis therapy even in the quest for faster growth and, in so doing telestroke may function as a model to relax the criteria for the inclusion or exclusion for thrombolysis in hypertensive stroke patients.

### Data collection

We used structured data collected from electronic health records of patients presenting acute ischemic stroke with history of hypertension between January 2014 and June 2016 from the Greenville Health System Stroke Registry. The stroke registry contained data for telestroke and non telestroke patients with a primary diagnosis of ischemic stroke and has been described in our previous studies [13, 18, 31, 37–40]. All data for the patient’s demographics and clinical variables from both the telestroke and non telestroke patients were abstracted by a stroke nurse. Baseline clinical risk factors were retrieved from documented patients’ medical history. Data were collected for demographics (age and gender), admission date, medications, clinical diagnosis, prehospital care, pre-stroke and post-stroke ambulatory status, in-hospital procedures, past medical history. All data were scrutinized under quality control checks using established protocol to determine the quality of the data and to insure against several types of errors, including: errors in interpretation or coding, errors in data entry. All data including neuroimaging data were reviewed by a clinician, who determined whether the patient met the clinical case description of acute stroke. The events were categorized as ischemic stroke, transient ischemic attack, intracerebral hemorrhage, or subarachnoid hemorrhage according to descriptions of stroke from the Classification of Cerebrovascular Diseases III [41]. Patients with missing brain imaging data or no information for times of receiving rtPA, and stroke patients with incidence hypertension without medical records were all excluded. In addition, we excluded data from patients that received endovascular therapy to maintain homogeneity of the data. Patients with a history of hypertension have been described in our previous study [23]. This is based on the guideline for the management of hypertension pre and post stroke according to Joint National Committee (JNC7) guideline, stroke with hypertension (stages 1;SBP; 140–159, DBP 90–99 and stage 2;SBP,> 160, DBP; or > 100) should be managed to levels or 130/80 mmHg or lower [42].

In addition, we collected data on symptom onset time and the admission to Emergency Department (ED) for both telestroke and non telestroke hypertensive stroke patients. We identified patients directly admitted to the ED or with emergency medical services (EMS). Patients with indirect admission by being transferred to the ED in the telestroke or non telestroke from another hospital were also identified. Onset time referred to the time that the patient first presented with neurological disorder or the last normal observation for unknown clinical conditions. We collected baseline information for NIHSS score, pre-rtPA systolic and diastolic blood pressure, and the presence of comorbid risk factors, including a history of diabetes mellitus, prior stroke or transient ischemic attack, and atrial fibrillation. Additional medical history including information on carotid artery stenosis, hypertension, prosthetic heart valve, renal insufficiency, smoking, sleep apnea, migraine, obesity, and peripheral vascular disease was collected. Information of demographics include age, sex, race, and ethnicity. Information of laboratory analysis was collected including total cholesterol, triglycerides, HDL, LDL, lipids, blood glucose, and creatine. Information on ambulation status prior to event, during and at discharge were also collected.

### Data analysis

All statistical analyses were performed utilizing SPSS Statistics Software version 15.0 (Chicago, IL) and *P* < 0.05 was used to establish statistical significance in all comparisons between groups. A univariate analysis was used to determine factors that were associated with inclusion or exclusion for rtPA in the population of hypertensive stroke patients. Descriptive statistics were calculated for the demographic and clinical characteristics of patients. All continuous variables are represented as mean ± standard deviation and comparisons between groups were determined using a Student’s T-test. All discrete variables are represented as number (percentage) and comparisons between groups were made using Pearson’s Chi-Squared analyses. Multivariate analysis was performed to determine demographic and clinical characteristics that were more associated with the telestroke subgroup utilizing a stepwise conditional logistic regression with *p* < 0.05. Univariate analysis was repeated to determine factors associated with inclusion for rtPA in the separate telestroke subgroup and non-telestroke subgroup for hypertensive stroke patients. Multivariate analysis using a stepwise conditional logistic regression with *p* < 0.05 was performed to determine demographic and clinical characteristics associated with exclusion from rtPA administration in the total study population as well as in the telestroke subgroup and non-telestroke subgroup. All stepwise regression models were assessed using Hosmer & Lemeshow test, Cox & Snell R^2^ and Classification Plots and ROC analysis. Multicollinearity of variables were assessed with variance inflation factor analysis to confirm independence of variables included in the regression model. We presented all results for our multivariate analysis as odds ratio estimates at 95% confidence interval and results were considered significant at *P* < 0.05.

## Results

A total of 434 stroke patients with hypertension were admitted to the non telestroke while 267 were admitted in the telestroke. Comparisons between the baseline demographic and clinical characteristics of telestroke and non-telestroke patients are presented in Table 1. Compared to the non-telestroke control group, telestroke patients were younger (66.1 ± 13.5 vs. 70.1 ± 14.1), included more African-American or other minority group individuals (19.1% vs. 12.2%), and have a higher body mass index (30.8 ± 7.3 vs. 28.2 ± 6.7). Prior to admission, telestroke patients had lower rates of atrial fibrillation or atrial flutter (11.6% vs. 27.2%) and were less likely to have had a previous stroke (24.7% vs. 32.0%), but more likely to have diabetes (46.1% vs. 38.0%), depression (18.0% vs 0.2%), hormone replacement therapy (3%0 vs 0.7%) and more likely to be currently medicated for diabetes (37.1% vs. 27.4%). Initial labs at the time of presentation showed that telestroke patients tended to have lower creatinine (1.2 ± 0.8 vs. 1.4 ± 1.1), lower INR (1.0 ± 0.2 vs. 1.1 ± 0.5), lower heart rate (78.2 ± 15.9 vs. 82.7 ± 18.5), systolic blood pressure (148.3 ± 24 vs 155.9 ± 30.9), and diastolic blood pressure (79.8 ± 16.3 vs. 83.6 ± 20.6). Telestroke patients also had a better ambulatory status prior to admission, at the time of presentation, and at discharge, were more likely to receive rtPA (89.1% vs. 38.7%) and were more likely to experience an improved ambulatory status at discharge relative to presentation (70.4% vs. 58.1%).

**Table 1:** Demographic factors and clinical characteristics of acute ischemic stroke patients with a history of hypertension divided by telestroke status

Characteristic	Non-Telestroke (*N* = 434)	Telestroke (*N* = 267)	*P*-Value
Patient Age in Years			
Mean ± SD	70.1 ± 14.1	66.1 ± 13.5	< 0.001*
Age Group: No. (%)			
< 50 years	45 (10.4)	31 (11.6)	0.002*
50–59	56 (12.9)	47 (17.6)	
60–69	88 (20.3)	76 (28.5)	
70–79	118 (27.2)	67 (25.1)	
≥ 80	127 (29.3)	46 (17.2)	
Gender: No. (%)			
Male	203 (46.8)	139 (52.1)	0.174
Female	231 (53.2)	128 (47.9)	
Race: No. (%)			
Caucasian	346 (79.7)	209 (78.3)	0.010*
African-American	50 (11.5)	46 (17.2)	
Other	3 (0.7)	5 (1.9)	
Hispanic Ethnicity: No. (%)	5 (1.2)	6 (2.2)	0.257
Body Mass Index			
Mean ± SD	28 .2 ± 6.7	30.8 ± 7.3	< 0.001*
Medical History: No. (%)			
Atrial Fib/Flutter	118 (27.2)	31 (11.6)	< 0.001*
Carotid Artery Stenosis	25 (5.8)	16 (6)	0.899
Congestive Heart Failure	61 (14.1)	33 (12.4)	0.522
Coronary Artery Disease	173 (39.9)	106 (39.7)	0.966
Depression	1 (0.2)	48 (18)	< 0.001*
Diabetes	165 (38)	123 (46.1)	0.035*
Dyslipidemia	259 (59.7)	168 (62.9)	0.393
Family History of Stroke	35 (8.1)	32 (12)	0.086
Hormone Replacement Therapy	3 (0.7)	8 (3)	0.017*
Migraine	7 (1.6)	8 (3)	0.219
Obesity	36 (8.3)	25 (9.4)	0.626
Peripheral Vascular Disease	(0)	(0)	
Previous Stroke	139 (32)	66 (24.7)	0.039*
Previous TIA	59 (13.6)	32 (12)	0.538
Prosthetic Heart Valve	9 (2.1)	1 (0.4)	0.065
Renal Insufficiency	33 (7.6)	17 (6.4)	0.537
Sleep Apnea	0 (0)	12 (4.5)	< 0.001*
Smoking	115 (26.5)	67 (25.1)	0.681
Substance Abuse	17 (3.9)	7 (2.6)	0.36
Initial NIH Stroke Scale			
Mean ± SD	10.7 ± 8.5	9.5 ± 8.2	0.076
Initial Labs & Vitals			
Total Cholesterol	164.9 ± 48.9	166.6 ± 42.4	0.651
Triglycerides	133.2 ± 89.5	147.7 ± 103	0.063
HDL	40.9 ± 13.9	40.2 ± 12.7	0.547
LDL	98.1 ± 37.1	100.3 ± 35.3	0.443
Lipids	6.4 ± 1.7	6.5 ± 1.7	0.659
Blood Glucose	151.1 ± 86.4	138.9 ± 72.4	0.058
Creatinine	1.4 ± 1.1	1.2 ± 0.8	0.006*
INR	1.1 ± 0.5	1 ± 0.2	< 0.001*
Heart Rate	82.7 ± 18.5	78.2 ± 15.9	0.001*
Systolic Blood Pressure	155.9 ± 30.9	148.3 ± 24	< 0.001*
Diastolic Blood Pressure	83.6 ± 20.6	79.8 ± 16.3	0.007*
Medications Prior to Admission: No. (%)			
Antiplatelet or Anticoagulant	256 (59)	151 (56.6)	0.526
Antihypertensive	369 (85)	233 (87.3)	0.408
Cholesterol Reducer	222 (51.2)	148 (55.4)	0.27
Diabetic Medication	119 (27.4)	99 (37.1)	0.007*
Ambulation Status Prior to Event: No. (%)			
Ambulate Independently	377 (86.9)	252 (94.4)	0.012*
Ambulate With Assistance	22 (5.1)	4 (1.5)	
Unable to Ambulate	19 (4.4)	7 (2.6)	
Not Documented	16 (3.7)	4 (1.5)	
Ambulation Status on Admission: No. (%)			
Ambulate Independently	60 (13.8)	55 (20.6)	0.005*
Ambulate With Assistance	63 (14.5)	53 (19.9)	
Unable to Ambulate	162 (37.3)	72 (27)	
Not Documented	149 (34.3)	87 (32.6)	
Ambulation Status on Discharge: No. (%)			
Ambulate Independently	162 (37.3)	132 (49.4)	< 0.001*
Ambulate With Assistance	128 (29.5)	84 (31.5)	
Unable to Ambulate	102 (23.5)	33 (12.4)	
Not Documented	42 (9.7)	18 (6.7)	
First Care Received: No. (%)			
Emergency Department	395 (91)	78 (29.2)	< 0.001*
Direct Admission	39 (9)	189 (70.8)	
rtPA Administration	168 (38.7)	238 (89.1)	< 0.001*
Improved Ambulation	252 (58.1)	188 (70.4)	0.001*

Continuous variables are represented as Mean ± S.D. and comparisons between groups are made with a Student’s T Test. Discrete variables are represented as Count (Percent Frequency) and comparisons between groups were made using Pearson’s Chi-Squared

*P<0.05

Clinical characteristics associated with the inclusion and exclusion for rtPA for patients with a history of hypertension in the telestroke and non-telestroke cohorts are presented in Table 2. Of the 434 that were admitted in the non telestroke 61.3% were excluded from rtPA while 38.7% received rtPA. For the telestroke, 10.9% were excluded while 89.1% hypertensive stroke patients received rtPA. In the non-telestroke, patients who received rtPA were younger than patients who did not receive rtPA (68 ± 14.3 vs. 71.4 ± 13.9). Patients who received rtPA outside of a telestroke network presented with lower rates of carotid artery stenosis (2.4% vs. 7.9%), congestive heart failure (9.5% vs. 16.9%), previous stroke (23.2% vs. 37.6%), prosthetic heart valve (0% vs. 3.4%), renal insufficiency (3.0% vs. 10.5%), and lower blood glucose level (138.6 ± 68.7 vs. 158.9 ± 95.1). Patients treated with rtPA outside of the telestroke network also presented with a lower creatinine (1.2 ± 0.5 vs. 1.5 ± 1.4), and INR (1.1 ± 0.1 vs. 1.2 ± 0.6). Patients who received rtPA were more likely to experience improvement in their ambulatory status from presentation to discharge (65.5% vs. 53.4%). Within the telestroke subgroup there were fewer statistically significant differences between patients who received rtPA and patients who were excluded from rtPA. Patients who received rtPA were more likely to have a family history of stroke (13.4% vs 0%).

**Table 2:** Clinical characteristics, medical history, and presenting symptoms of acute ischemic stroke patients with a history of hypertension stratified by rtPA status and telestroke status

Characteristic	Non-Telestroke			Telestroke		
	No rtPA (*N* = 266)	rtPA (*N* = 168)	*P*-Value	No rtPA (*N* = 29)	rtPA (*N* = 238)	*P*-Value
Patient Age in Years						
Mean ± SD	71.4 ± 13.9	68 ± 14.3	0.015*	65.9 ± 13.6	66.1 ± 13.5	0.932
Age Group: No. (%)						
< 50 years	27 (10.2)	18 (10.7)	0.007*	2 (6.9)	29 (12.2)	0.268
50–59	25 (9.4)	31 (18.5)		9 (31)	38 (16)	
60–69	47 (17.7)	41 (24.4)		7 (24.1)	69 (29)	
70–79	83 (31.2)	35 (20.8)		5 (17.2)	62 (26.1)	
≥ 80	84 (31.6)	43 (25.6)		6 (20.7)	40 (16.8)	
Gender: No. (%)						
Male	121 (45.5)	82 (48.8)	0.499	15 (51.7)	124 (52.1)	0.969
Female	145 (54.5)	86 (51.2)		14 (48.3)	114 (47.9)	
Race: No. (%)						
Caucasian	207 (77.8)	139 (82.7)	0.451	22 (75.9)	187 (78.6)	0.725
African-American	33 (12.4)	17 (10.1)		4 (13.8)	42 (17.6)	
Other	3 (1.1)	0 (0)		1 (3.4)	4 (1.7)	
Hispanic Ethnicity: No. (%)	3 (1.1)	2 (1.2)	0.952	0 (0)	6 (2.5)	0.387
Body Mass Index						
Mean ± SD	28 ± 6.7	28.6 ± 6.7	0.312	31.5 ± 7.2	30.7 ± 7.3	0.596
Medical History: No. (%)						
Atrial Fib/Flutter	81 (30.5)	37 (22)	0.055	4 (13.8)	27 (11.3)	0.698
Carotid Artery Stenosis	21 (7.9)	4 (2.4)	0.016*	1 (3.4)	15 (6.3)	0.541
Congestive Heart Failure	45 (16.9)	16 (9.5)	0.031*	6 (20.7)	27 (11.3)	0.149
Coronary Artery Disease	103 (38.7)	70 (41.7)	0.542	11 (37.9)	95 (39.9)	0.837
Depression	1 (0.4)	0 (0)	0.426	4 (13.8)	44 (18.5)	0.534
Diabetes	103 (38.7)	62 (36.9)	0.704	16 (55.2)	107 (45)	0.297
Dyslipidemia	160 (60.2)	99 (58.9)	0.8	16 (55.2)	152 (63.9)	0.36
Family History of Stroke	22 (8.3)	13 (7.7)	0.843	0 (0)	32 (13.4)	0.035*
Hormone Replacement Therapy	3 (1.1)	0 (0)	0.167	0 (0)	8 (3.4)	0.316
Migraine	2 (0.8)	5 (3)	0.073	1 (3.4)	7 (2.9)	0.88
Obesity	93 (35)	58 (34.5)	0.926	15 (51.7)	137 (57.6)	0.549
Peripheral Vascular Disease	22 (8.3)	14 (8.3)	0.982	1 (3.4)	24 (10.1)	0.247
Previous Stroke	100 (37.6)	39 (23.2)	0.002*	7 (24.1)	59 (24.8)	0.939
Previous TIA	32 (12)	27 (16.1)	0.231	4 (13.8)	28 (11.8)	0.751
Prosthetic Heart Valve	9 (3.4)	0 (0)	0.016*	0 (0)	1 (0.4)	0.727
Renal Insufficiency	28 (10.5)	5 (3)	0.004*	1 (3.4)	16 (6.7)	0.495
Sleep Apnea	0 (0)	0 (0)	N/A	1 (3.4)	11 (4.6)	0.773
Smoking	62 (23.3)	53 (31.5)	0.058	9 (31)	58 (24.4)	0.434
Substance Abuse	11 (4.1)	6 (3.6)	0.768	1 (3.4)	6 (2.5)	0.768
Initial NIH Stroke Scale						
Mean ± SD	10.1 ± 9	11.5 ± 7.9	0.124	9.2 ± 8.3	9.5 ± 8.3	0.851
Initial Labs & Vitals						
Total Cholesterol	165 ± 52.8	164.7 ± 43.2	0.956	180.7 ± 50.2	164.9 ± 41.2	0.068
Triglycerides	132 ± 89.5	134.9 ± 89.8	0.759	147.4 ± 72.9	147.7 ± 106.1	0.987
HDL	40.7 ± 14.6	41.1 ± 13	0.834	42 ± 14.1	40 ± 12.5	0.44
LDL	97.9 ± 36.7	98.3 ± 37.8	0.91	111.6 ± 45.3	99 ± 33.8	0.08
Lipids	6.5 ± 1.8	6.3 ± 1.5	0.215	6.7 ± 1.9	6.4 ± 1.7	0.539
Blood Glucose	158.9 ± 95.1	138.6 ± 68.7	0.011*	164.4 ± 113.6	135.9 ± 65.4	0.203
Creatinine	1.5 ± 1.4	1.2 ± 0.5	0.001*	1.4 ± 1.8	1.1 ± 0.5	0.356
INR	1.2 ± 0.6	1.1 ± 0.1	0.001*	1.1 ± 0.4	1 ± 0.1	0.588
Heart Rate	83.4 ± 19	81.5 ± 17.7	0.284	76 ± 13.9	78.4 ± 16.2	0.444
Systolic Blood Pressure	154.5 ± 30.7	158.3 ± 31.2	0.217	152 ± 24.9	147.8 ± 23.8	0.38
Diastolic Blood Pressure	82.6 ± 21	85.2 ± 19.8	0.214	78.8 ± 13.8	79.9 ± 16.6	0.721
Medications Prior to Admission: No. (%)						
Antiplatelet or Anticoagulant	162 (60.9)	94 (56)	0.307	18 (62.1)	133 (55.9)	0.526
Antihypertensive	222 (83.5)	147 (87.5)	0.25	25 (86.2)	208 (87.4)	0.856
Cholesterol Reducer	137 (51.5)	85 (50.6)	0.854	18 (62.1)	130 (54.6)	0.446
Diabetic Medication	74 (27.8)	45 (26.8)	0.814	11 (37.9)	88 (37)	0.92
Ambulation Status Prior to Event: No. (%)						
Ambulate Independently	218 (82)	159 (94.6)	0.002*	25 (86.2)	227 (95.4)	0.246
Ambulate With Assistance	18 (6.8)	4 (2.4)		1 (3.4)	3 (1.3)	
Unable to Ambulate	16 (6)	3 (1.8)		2 (6.9)	5 (2.1)	
Not Documented	14 (5.3)	2 (1.2)		1 (3.4)	3 (1.3)	
Ambulation Status on Admission: No. (%)						
Ambulate Independently	46 (17.3)	14 (8.3)	< 0.001*	11 (37.9)	44 (18.5)	0.023*
Ambulate With Assistance	48 (18)	15 (8.9)		4 (13.8)	49 (20.6)	
Unable to Ambulate	95 (35.7)	67 (39.9)		10 (34.5)	62 (26.1)	
Not Documented	77 (28.9)	72 (42.9)		4 (13.8)	83 (34.9)	
Ambulation Status on Discharge: No. (%)						
Ambulate Independently	94 (35.3)	68 (40.5)	0.45	15 (51.7)	117 (49.2)	0.736
Ambulate With Assistance	78 (29.3)	50 (29.8)		7 (24.1)	77 (32.4)	
Unable to Ambulate	64 (24.1)	38 (22.6)		4 (13.8)	29 (12.2)	
Not Documented	30 (11.3)	12 (7.1)		3 (10.3)	15 (6.3)	
First Care Received: No. (%)						
Emergency Department	242 (91)	153 (91.1)	0.973	18 (62.1)	60 (25.2)	< 0.001*
Direct Admission	24 (9)	15 (8.9)		11 (37.9)	178 (74.8)	
Improved Ambulation	142 (53.4)	110 (65.5)	0.013*	20 (69)	168 (70.6)	0.857

Continuous variables are represented as Mean ± S.D. and comparisons between groups are made with a Student’s T Test. Discrete variables are represented as Count (Percent Frequency) and comparisons between groups were made using Pearson’s Chi-Squared

*P<0.05

**Fig. 1:**
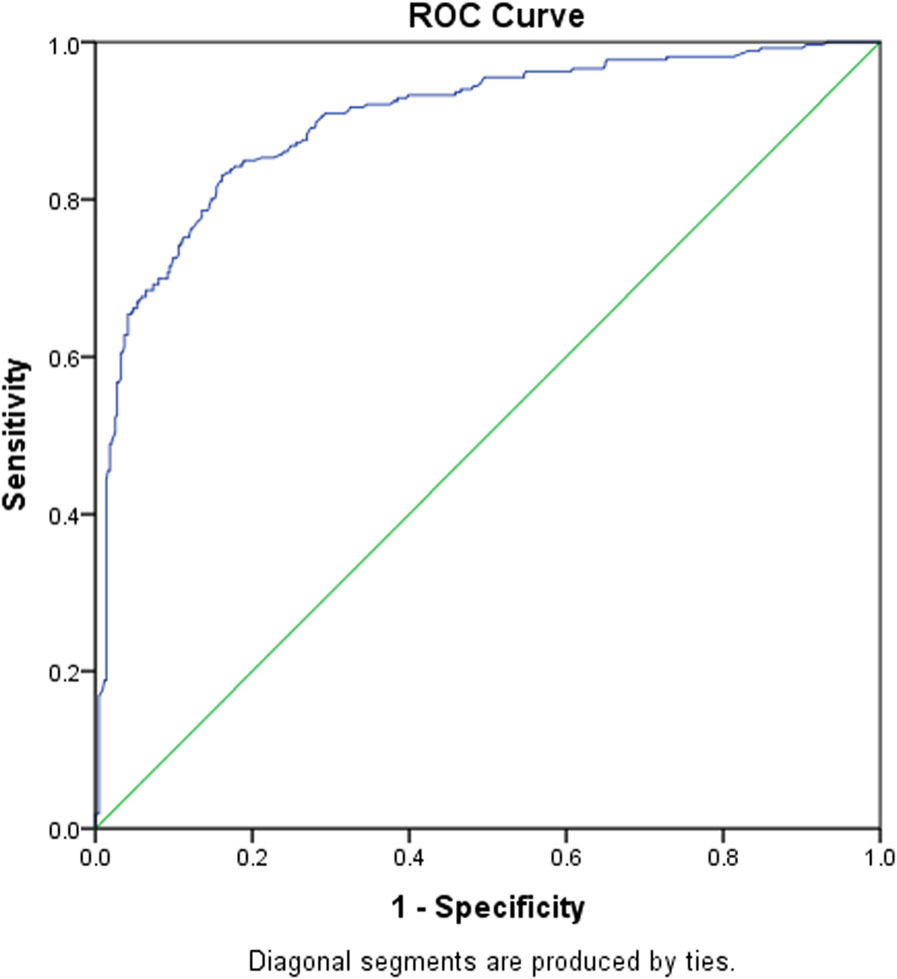
ROC curve to analyze the predictive power of the logistic regression presented in Table 2. AUROC = 0.900 (0.875–0.924, *P* < 0.05)

**Fig. 2:**
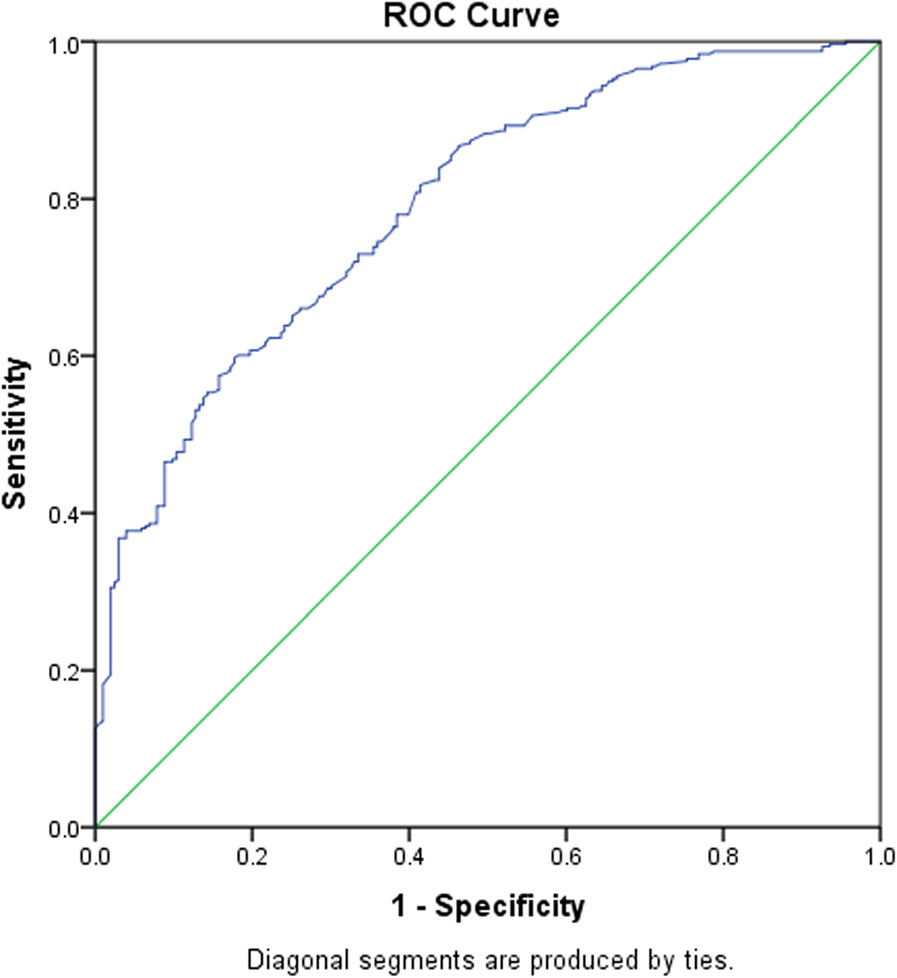
ROC curve to analyze the predictive power of the logistic regression presented in Table 4. AUROC = 0.792 (0.753–0.830, *P* < 0.05)

Following the use of multivariate analysis to adjust for the cofounding effects of comorbidities in the telestroke and non-telestroke (Table 3), patients that present with obesity (OR = 2.351, 95% CI, 1.352–4.087, *P* = 0.002) were directly admitted for treatment (OR = 32.855, 95% CI, 15.706–68.727, *P* < 0.001) and received rtPA (OR = 5.199, 95% CI, 2.647–10.211, *P* < 0.001) have higher odds of being associated with the telestroke, while higher systolic blood pressure (OR = 0.987, 95% CI, 0.977–0.997, *P* = 0.01) was associated with the non telestroke. The Receiver Operating Characteristics (ROC) curve for the predictive power of the regression model is presented in Fig. 1. The discriminating capability of the model was very good as shown by the ROC curve, with area under the curve (AUROC) of 0.900 (95% CI, 0.875–0.924, *P* < 0.001). Further adjusted analysis was performed focusing on the whole stroke population (Table 4). This analysis determined clinical and demographic factors that were associated with inclusion for rtPA, irrespective of whether patients were treated in the telestroke or non telestroke setting. The results indicate that telestroke as a variable was the strongest predictor of rtPA administration (OR = 5.204, 95% CI, 2.582–10.492, *P* < 0.001), followed by a direct admission (OR = 4.557, 95% CI, 1.772–11.721, *P* = 0.002), and higher NIH stroke scale score (OR = 1.046, 95% CI, 1.016–1.076, *P* = 0.002). A higher INR, (OR = 0.203, 95% CI, 0.06–0.691, *P* = 0.011), history of previous stroke (OR = 0.476, 95% CI, 0.293–0.775, *P* = 0.003), and renal insufficiency, (OR = 0.351, 95% CI, 0.143–0.858, *P* = 0.022), were predictive of exclusion of stroke patients with hypertension from rtPA. As shown in Fig. 2, the predictive power of the logistic regression was strong. The area under the curve (AUROC) is 0.792 (95% CI, 0.753–0.830, *P* < 0.05).

**Table 3:** A stepwise regression model to elucidate clinical factors more associated with acute ischemic stroke patients presenting via telestroke

	B Value	Adj. Odds Ratio	Wald	*P* Value
Systolic Blood Pressure	−0.013	0.987 (0.977–0.997)	6.549	0.01*
Obesity	0.855	2.351 (1.352–4.087)	9.183	0.002*
Direct Admission	3.492	32.855 (15.706–68.727)	86.004	< 0.001*
rtPA Administration	1.649	5.199 (2.647–10.211)	22.916	< 0.001*
Constant	−1.003	0.367	1.401	0.237

Positive B values (Adj, OR > 1) denote variables more associated with telestroke patients while negative B values (Adj. OR < 1) denote variables more associated with non-telestroke patients. Multicollinearity and interactions among independent variables were checked. Hosmer-Lemeshow test (*P* = 0.089), Cox & Snell (R^2^ = 0.432), Classification table (overall correctly classified percentage = 85.3%) were applied to check the model fitness

*P<0.05

**Table 4:** A stepwise regression model to elucidate clinical factors more associated rtPA inclusion in the total study population

	B Value	Adj. Odds Ratio	Wald	*P* Value
NIH Stroke Scale Score	0.045	1.046 (1.016–1.076)	9.320	0.002*
INR	−1.595	0.203 (0.06–0.691)	6.502	0.011*
Previous Stroke	−0.742	0.476 (0.293–0.775)	8.934	0.003*
Renal Insufficiency	−1.048	0.351 (0.143–0.858)	5.271	0.022*
Direct Admission	1.517	4.557 (1.772–11.721)	9.900	0.002*
Telestroke	1.650	5.204 (2.582–10.492)	21.262	< 0.001*
Constant	1.490	4.439	4.731	0.03*

Positive B values (Adj, OR > 1) denote variables more associated with rtPA inclusion while negative B values (Adj. OR < 1) denote variables more associated with rtPA exclusion. Multicollinearity and interactions among independent variables were checked. Hosmer-Lemeshow test (*P* = 0.006), Cox & Snell (R^2^ = 0.260), classification table (overall correctly classified percentage = 75.3%) were applied to check the model fitness

*P<0.05

**Fig. 3:**
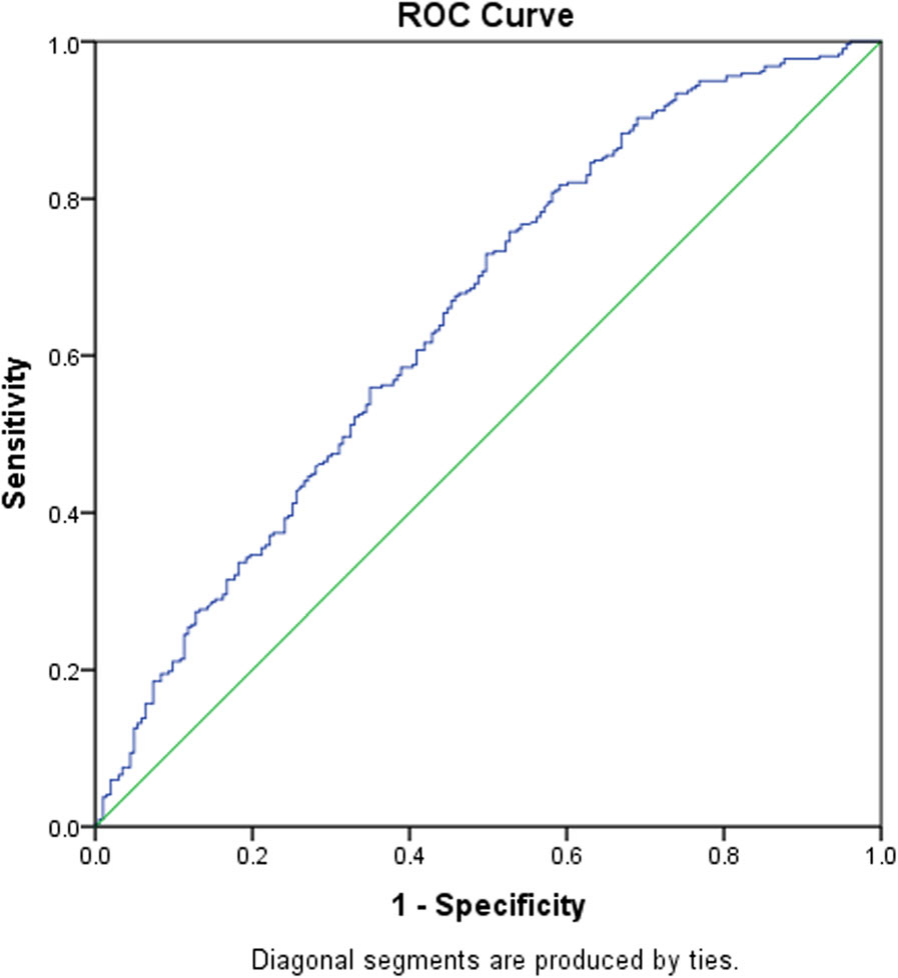
ROC curve to analyze the predictive power of the logistic regression presented in Table 5. AUROC = 0.650 (0.602–0.699, *P* < 0.05)

**Fig. 4:**
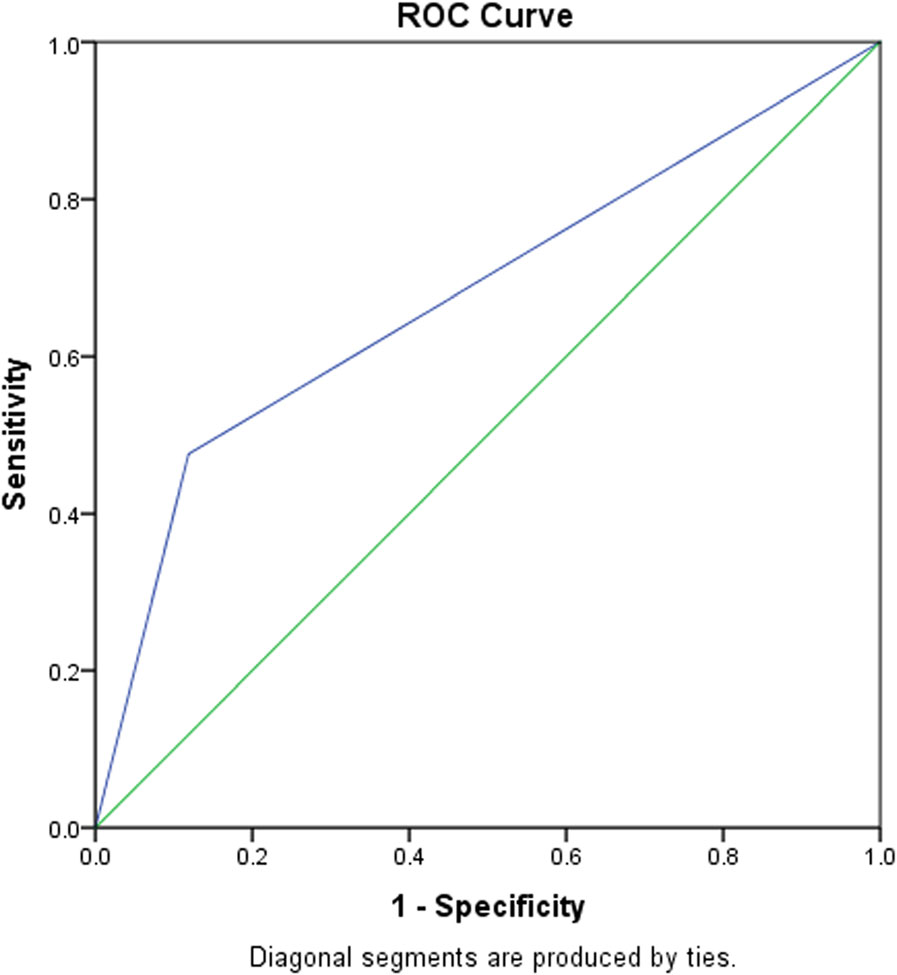
ROC curve to analyze the predictive power of the logistic regression presented in Table 6. AUROC = 0.678 (0.639–0.718, *P* < 0.05)

In the non-telestroke subgroup (Table 5), higher NIH stroke scale score (OR = 1.059, 95% CI, 1.025–1.093, *P* < 0.001) and history of coronary artery disease (OR = 2.003, 955 CI, 1.16–3.457, *P* = 0.013) were associated with rtPA administration while increasing age (OR = 0.979, 95% CI, 0.961–0.996, *P* = 0.017), higher INR (OR = 0.146, 95% CI, 0.032–0.665, *P* = 0.013), history of previous stroke (OR = 0.39, 95% CI, 0.223–0.68, *P* = 0.001), and renal insufficiency (OR = 0.153, 95% CI, 0.046–0.508, *P* = 0.002) are associated with rtPA exclusion. The ROC curve reveals a strong prediction of the logistic regression model (Fig. 3), AUROC = 0.650 (95% CI, 0.602–0.699, *P* < 0.005). An adjusted analysis for the telestroke subgroup (Table 6), reveals that only direct admission is associated with rtPA administration, (OR = 4.083, 95% CI, 1.322–12.611, *P* = 0.014), and the predictive model power of the logistic regression was strong (Fig. 4), AUROC = 0.678 (95% CI, 0.639–0.718, *P* < 0.05).

**Table 5:** A stepwise regression model to elucidate clinical factors more associated rtPA inclusion in the non-telestroke population

	B Value	Adj. Odds Ratio	Wald	*P* Value
Increasing Age	−0.022	0.979 (0.961–0.996)	5.726	0.017*
NIH Stroke Scale Score	0.057	1.059 (1.025–1.093)	12.190	< 0.001*
INR	−1.927	0.146 (0.032–0.665)	6.180	0.013*
Coronary Artery Disease	0.695	2.003 (1.16–3.457)	6.222	0.013*
Previous Stroke	−0.942	0.39 (0.223–0.68)	10.984	0.001*
Renal Insufficiency	−1.879	0.153 (0.046–0.508)	9.387	0.002*
Constant	3.122	22.700	9.628	0.002*

Positive B values (Adj, OR > 1) denote variables more associated with rtPA inclusion while negative B values (Adj. OR < 1) denote variables more associated with rtPA exclusion. Multicollinearity and interactions among independent variables were checked. Hosmer-Lemeshow test (*P* = 0.854), Cox & Snell (R^2^ = 0.159), classification table (overall correctly classified percentage = 67%) were applied to check the model fitness

*P<0.05

**Table 6:** A stepwise regression model to elucidate clinical factors more associated rtPA inclusion in the telestroke population

	B Value	Adj. Odds Ratio	Wald	*P* Value
Direct Admission	1.407	4.083 (1.322–12.611)	5.979	0.014*
Constant	1.569	4.800	20.363	< 0.001*

*P<0.05

## Discussion

In a population of hypertensive stroke patients, we found patients that present with obesity, who are directly admitted to the hospital, and received rtPA have higher odds of being associated with the telestroke, while those with a higher systolic blood pressure were associated with the non telestroke. After adjusting for comorbidities without sorting by telestroke and non telestroke, we found that irrespective of whether patients were treated in the telestroke or non telestroke, the telestroke represents the strongest predictor of rtPA administration followed by a direct admission and NIH stroke scale scores. However, a higher INR, history of previous stroke and renal insufficiency were predictors of exclusion of hypertensive stroke patients from rtPA.

In the univariate analysis for the non telestroke, a higher NIH stroke scale score and history of coronary artery disease were associated with inclusion for rtPA while increasing age, higher INR, history of previous stroke, and renal insufficiency were all associated with rtPA exclusion. Following an adjustment for the baseline clinical variables in the telestroke subgroup, direct admission was the only factor associated with rtPA administration with a strong predictive power in the regression model. Clearly, the higher proportion of clinical risk factors associated with exclusion in the non telestroke when compared with the telestroke is not unconnected with the exclusion of more hypertensive stroke patients from rtPA; because though the non telestroke admitted more patients (62% vs 38%) when compared with the telestroke, more patients (61.3% vs 38.7%) were excluded from rtPA. We observed that the telestroke admitted fewer patients, excluded 11% and administered thrombolysis therapy to 89% of hypertensive stroke patients. It is possible that the telestroke technology provides a real-world clinical experience with less stringent or restrictive exclusion criteria focused more on benefit-to-risk ratio for thrombolysis therapy. The fact that the non telestroke group had more clinical risk factors associated with exclusion from thrombolysis indicates that stricter criteria were used when compared with the telestroke group, which had less stringent criteria on an individual basis. In a whole stroke hypertensive population telestroke was the strongest predictor of rtPA administration, followed by a direct admission and higher NIH stroke scale score respectively. Our model predicted a direct association of telestroke with the highest odds for the inclusion of hypertensive stroke patients for thrombolysis therapy. Other studies have indirectly linked the telestroke technology with the improvement of stroke care in rural health care centers [13], increased rate of rtPA administration [38, 43] and improvement of the timeliness of rtPA administration [39, 40]. Our finding of a strong association of telestroke with thrombolysis may reflect the fast response for the early recommendation of administration of rtPA in the hub station and faster administration in the spoke station resulting in significant increases in the rates of rtPA utilization at the spoke hospitals. There is a growing evidence that telemedicine services offer hypertensive patients the access to diagnostic measures [41, 44] that might not be available with usual bedside evaluation care. This indicates that telemedicine has the ability to deconstruct the typical traditional model in the standard clinical consultation [29]. This might impact the management of hypertension in ischemic stroke beyond the use of antiHTN in the telestroke with some form of additional support compared with usual care not common in the non telestroke. In this context, the telestroke could be a part of the solution to improve the rate of thrombolysis in hypertensive stroke patients.

In the univariate analysis, the effect of direct admission was not significant in both the telestroke and non telestroke settings. Following an adjusted analysis for both the telestroke and non telestroke, a direct admission of patients was associated with thrombolysis in the telestroke setting. The significant association of direct admission with thrombolysis was sustained in the whole stroke population following adjustment for baseline demographic and clinical risk factors. In general, direct admission is known to be associated with shorter onset-to-needle time and better outcome in patients with acute ischemic stroke undergoing thrombolysis [45–50]. This is because a direct admission to the telestroke could reduce the door-to-needle time for thrombolysis to within an hour in more than 70% of acute ischemic stroke patients and less than 50 min in more than 45% of stroke patients [51–53]. Many methods have been proposed to reduce door-to-needle time for thrombolysis, including in-hospital system-level centralized telestroke care [10, 13, 16, 54–58]. Therefore, it is possible that in hypertensive stroke patients, telestroke may be associated with improved thrombolysis therapy use by facilitating a direct admission that improves the time to receive thrombolysis therapy. Such a time improvement could be due to the reduction of the time between hub stroke neurologist’s and spoke clinician’s involvement in the management, treatment, and implementation of the protocol to mix rtPA and administer thrombolysis to hypertensive stroke patients.

Our finding that INR, previous stroke and increased age (> 80 years) are associated with exclusion of hypertensive stroke patients from rtPA have been reported as exclusion variables in acute ischemic stroke patients [3, 59–66]. In general, existing guidelines regarding INR results are intended to prevent hemorrhages and, specifically intracranial hemorrhage which is the most dreaded complication of rtPA in patients taking oral anticoagulants. In particular, the AHA/ASO guidelines [67] recommend that, the administration of rtPA can be initiated before results of coagulation tests or platelet counts are available, unless a bleeding is observed after the patient had already received heparin or other anticoagulants. However, if rtPA protocol has been initiated and the results of the coagulation tests or platelet counts indicate that INR > 1.7 or a PT > 15 s by local protocol of rtPA administration, then the procedure should be stopped [68]. This might have contributed to the exclusion of more patients in the non telestroke in our current study.

We found that hypertensive stroke patients with age > 80 years are more likely to be excluded more from rtPA in the non telestroke setting. Observational studies aimed at monitoring the safety of rtPA provided comparative results on stroke outcome between patients > 80 years and those < 80 years [63, 69–72]. Findings reveal that > 80 years are less likely to achieve good outcomes compared with < 80 years. Most of the observational studies evaluated age differences between older and younger patients receiving rtPA. In our adjusted analysis, more hypertensive stroke patients > 80 years are more likely to be excluded from rtPA compared with > 80 years that received rtPA in the non telestroke. In general, patients with an elevated INR and a previous stroke within the last 3 months are associated with contraindications for rtPA in other studies [38, 64, 73–77]. Therefore, the combined effect of old age coupled with elevated INR and a previous stroke within the last 3 months played a role in the exclusion criteria in acute ischemic stroke patients with incidence hypertension in the current study.

There are limitations in this study. This is a single healthcare system initiative with retrospective data collection. Therefore, there is the tendency for selection bias because of the lack of an experimental design that allows for randomization, especially at telestroke or non telestroke settings. For this reason, our finding cannot be generalized to other hospital settings. Since our data is pretreatment, and post treatment only until discharge from the hospital, it did not include longitudinal data for a follow-up to determine the effect of direct admission on post rtPA outcome. Moreover, information about the management of hypertension and the time for direct and indirect admission was not included in our analysis, preventing comparison of onset-to-door time between indirect and direct admission. The number ischemic stroke patients that needed antihypertensive treatment before rtPA were not included. However, our model associated telestroke with thrombolysis in providing clinical practice with less restrictive exclusions focused more on benefit-to-risk ratio for thrombolysis therapy in stroke patients with hypertension.

## Conclusion

In summary, telestroke was the strongest predictor of rtPA administration, followed by a direct admission and higher NIH stroke scale score. More studies are necessary to determine how identified exclusion risk factors in the non telestroke setting can be improved, including a direct transfer of hypertensive stroke patients to health care centers with a direct access to thrombolysis.

## Abbreviations

AHA: American Heart Association
AUROC: Area under the curve
CAD: Coronary artery disease
CHF: Congestive heart failure
CI: Confidence interval
GHS: Greenville Health System
GWTG: Get with the guideline
MAP: Mean arterial pressure
NIH scores: National Institute of Health scores
OR: Adjusted odd ratio
PVD: Peripheral vascular disease
ROC: Receiver operator characteristic
rtPA: Recombinant tissue plasminogen activator
TIA: Trans ischemic attack
